# Nitrogen Addition and Heterotroph Exclusion Affected Plant Species Diversity–Biomass Relationship by Affecting Plant Functional Traits

**DOI:** 10.3390/plants13020258

**Published:** 2024-01-16

**Authors:** Xile Xu, Luping Yang, Kai Shen, Huijuan Cao, Yishi Lin, Jinliang Liu, Wenjuan Han

**Affiliations:** 1 College of Life and Environmental Science, Wenzhou University, Wenzhou 325035, China; 21451335046@stu.wzu.edu.cn (X.X.); 21461337123@stu.wzu.edu.cn (L.Y.); 21461337078@stu.wzu.edu.cn (K.S.); 22451335002@stu.wzu.edu.cn (H.C.); 22461337071@stu.wzu.edu.cn (Y.L.); jinliang.liu@foxmail.com (J.L.); 2 Zhejiang Provincial Key Laboratory for Water Environment and Marine Biological Resources Protection, Wenzhou University, Wenzhou 325035, China

**Keywords:** nitrogen addition, heterotroph removal, species identity, productivity, functional traits

## Abstract

(1) Background: Heterotrophs can affect plant biomass and alter species diversity–productivity relationships. However, these studies were conducted in systems with a low nitrogen (N) availability, and it is unclear how heterotroph removal affects the relationship between plant species diversity and productivity in different N habitats. (2) Methods: Three typical understory herbaceous plants were selected to assemble the plant species diversity (three plant species richness levels (1, 2, and 3) and seven plant species compositions), and the control, insecticide, fungicide, and all removal treatments were performed at each plant species diversity level in systems with or without N addition treatments. (3) Results: In systems without N addition, the insecticide treatment increased the plant aboveground biomass, total biomass, and leaf area, while the fungicide treatment reduced the plant belowground biomass, root length, and root tip number; the presence of *Bidens pilosa* increased the plant aboveground biomass. Similarly, the presence of *Bletilla striata* increased the plant belowground biomass and root diameter under each heterotroph removal treatment. In systems with N addition, all removal treatments reduced the plant belowground biomass and increased the plant leaf area; the presence of *B. pilosa* significantly increased the plant aboveground biomass, total biomass, and root length under each heterotroph removal treatment. The presence of *B. striata* significantly increased the plant belowground biomass and leaf area under insecticide and fungicide treatments. (4) Conclusions: Heterotroph removal alters the plant species diversity–biomass relationship by affecting the plant functional traits in systems with different N availabilities. The impact of biodiversity at different trophic levels on ecosystem functioning should be considered under the background of global change.

## 1. Introduction

Biodiversity is an important determinant of ecosystem function [1]. Plant species diversity (species richness and species identity) could enhance plant productivity through the selection effect and complementary effect [1,2,3,4]. However, most productivity measures did not account for the effects of heterotrophs on productivity [5,6,7]. Heterotrophs include herbivores, predators, scavengers, and pathogens. Previous studies showed that the removal of arthropods and foliar fungi increased plant biomass [8,9,10], while the removal of soil fungi increased the forb biomass in grassland systems [11]. Removing foliar fungi also increased the biomass of trees in forest systems [12]. Increasing the plant species diversity can increase the abundance of arthropods [13,14] or decrease the abundance of fungal pathogens [15], and the impact of heterotrophs on plant biomass may increase or decrease with an increasing plant species diversity. In addition, plant and microbial diversities may have complementary effects on nutrient cycling [16]; plant and herbivore diversities may have opposite effects on plant productivity [17]. Thus, considering the influence of heterotrophs on the plant species diversity–biomass relationship is necessary.

A few studies have concentrated on the impact of heterotroph removal on plant species diversity–biomass relationships [8,12]. In the grassland system, insecticide and fungicide treatments promoted the impact of plant diversity on productivity [8]. In forest systems, the positive relationship between tree species richness and productivity was eliminated when tree crowns were under a fungicide treatment [12]. However, all these studies were conducted in habitats with relatively low nitrogen (N) levels. The impact of heterotroph removal on the plant species diversity–biomass relationship in habitats with a high N level remains unclear.

Human activities such as industrial development and agricultural production have continuously increased atmospheric N deposition in the terrestrial ecosystem [18,19,20,21]. In habitats with a low N availability, N deposition could increase plant biomass [6,22,23]. Nevertheless, continuous N deposition could lead to N saturation, inhibited plant growth, and reduced plant biomass [24]. The increase in N availability in habitats may promote the growth of dominant plants, thereby increasing the selection effects [25]; it may also increase the complementary utilization of N by plants or promote interspecies interactions to enhance the complementary effect [7,26]. In addition, the increase in N availability in habitats may also alter the abundance of heterotrophs. For example, N addition reduced the number of soil microorganisms [27,28]. Thus, exploring the influence of heterotroph removal on plant species diversity–productivity relationships in high N habitats is necessary.

The functional traits of plant leaves and roots, such as the leaf area, root length, and root diameter, can reflect plants’ adaptability to the environment, their self-regulation ability in complex habitats, and their essential characteristics and effective utilization of resources [29]. Previous research showed that N deposition promoted the growth of the aboveground biomass of plants and specific leaf area [30,31], but excessive N would decrease the specific root length and belowground biomass [32]. The presence of herbivorous insects reduced the plant leaf area [33]. There was a direct interaction between soil microbial communities and roots; fungi and rhizobia could affect the ability of roots to capture nutrients from the soil [8,34].

To test how N addition and heterotroph exclusion affect the effect of plant species diversity on plant biomass, we conducted a three-factor (N addition, plant species diversity, and heterotrophic removal) control experiment, selecting three typical understory herbaceous plants, *Perilla frutescens*, *Bletilla striata*, and *Bidens pilosa*, to assemble the plant species diversity, and heterotroph removal was performed at each plant species diversity level. N deposition was simulated by N addition (10 g N m^−2^ yr^−1^). The plant above- and belowground biomasses and leaf and root functional traits of herbaceous plants were measured. We investigated the influence of heterotroph removal on plant biomass and functional traits in the system without/with N addition. We further investigated the effect of heterotroph removal on the plant diversity–biomass relationship in the system without/with N addition. We predicted that heterotroph exclusion and N addition may affect the plant species diversity–biomass relationship through the plant functional traits.

## 2. Results

### 2.1. Plant Biomass Responds to N Addition and Heterotroph Removal

**Figure 1:**
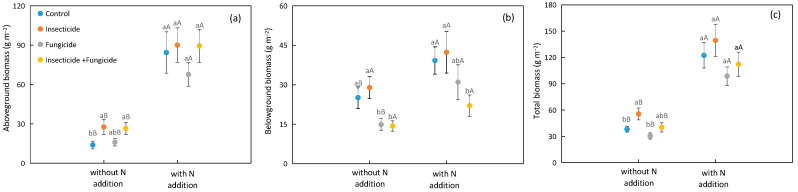
Difference in plant (**a**) aboveground, (**b**) belowground, (**c**) total biomass among heterotroph removal with or without N addition. Significant differences between systems without or with nitrogen addition were indicated in capital letters, and significant differences among heterotroph removal were indicated in lowercase letters. Each circle represents the average biomass of all species compositions under each heterotroph removal treatment. Blue: control; orange: insecticide; gray: fungicide; yellow: all removal.

N addition increased the plant biomass, with the plant aboveground, belowground, and total biomasses increased by 294.3%, 61.6%, and 178.5% on average, respectively (Figure 1). Under different heterotroph removal treatments, N addition improved the plant total and aboveground biomasses; under control and fungicide treatment groups, N addition also improved the plant belowground biomass (Figure 1).

In systems without N addition, the insecticide treatment increased the plant aboveground biomass by 98.9%, and all removal treatments increased the plant aboveground biomass by 90.3% relative to the control (Figure 1a); insecticide treatment also increased the plant total biomass by 45.9% relative to the control (Figure 1c). In systems with N addition, the study did not discover significant differences in the aboveground and total biomasses among various heterotroph removal treatments (Figure 1a,c). In systems with or without N addition, all removal treatments decreased the plant belowground biomass by 42.9% and 43.9% relative to the control, but insecticide treatment did not affect the plant belowground biomass (Figure 1b).

### 2.2. The Relationship between Plant Species Diversity and Plant Biomass

Plant species richness significantly improved the plant aboveground biomass, but plant belowground and total biomasses did not respond to plant species richness (Table S1). Plant species compositions also significantly affected the plant aboveground, belowground, and total biomasses (Table S1).

**Figure 2:**
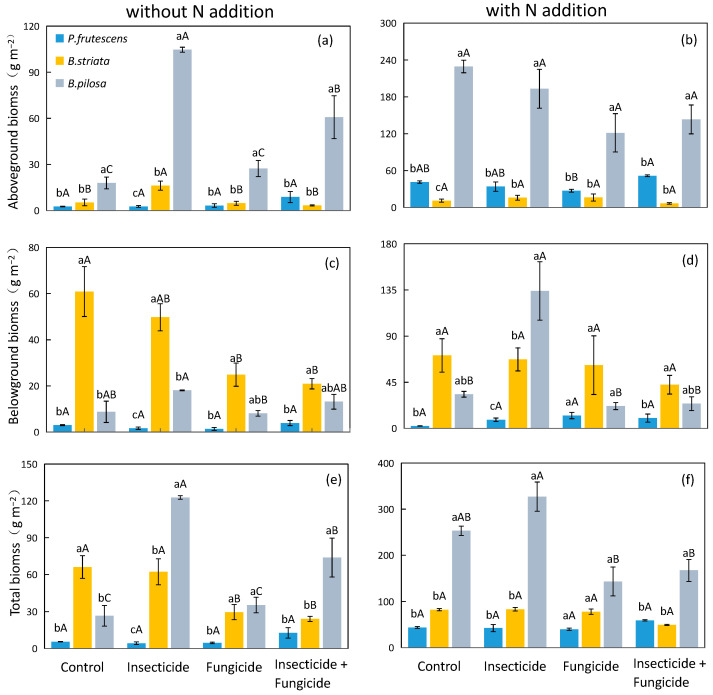
Difference in monoculture plant aboveground biomass (**a**), belowground biomass (**c**), and total biomass (**e**) in systems without N addition and plant aboveground biomass (**b**), belowground biomass (**d**), and total biomass (**f**) in systems with N addition among heterotroph exclusion with or without N addition. Significant differences between heterotroph removal groups were indicated in capital letters, and significant differences between plant species monocultures were indicated in lowercase letters. Blue bars: *P. frutescens* monoculture; yellow bars: *B. striata* monoculture; gray bars: *B. pilosa* monoculture.

In systems without N addition, the aboveground biomass of the *B. pilosa* monoculture was significantly higher than that of the *P. frutescens* monoculture and *B. striata* monoculture (Figure 2a), and the presence of *B. pilosa* significantly increased the plant aboveground and belowground biomasses under each heterotroph removal treatment (Table 1). It is worth noting that the plant total biomass was not affected by plant species identity in the control treatment. However, the plant total biomass was improved with fungicide treatment by 110.2% and all removal treatments by 155.8% when *B. pilosa* was present (Table 1).

In systems with N addition, the aboveground and total biomasses of the *B. pilosa* monoculture were significantly higher than those of the *P. frutescens* monoculture and *B. striata* monoculture (Figure 2b,f). Plant aboveground and total biomasses were improved when *B. pilosa* was present under each heterotroph removal treatment (Table 2). Significantly, the presence of *B. striata* decreased the aboveground biomass by 58.1% under control treatment, while the presence of *B. striata* did not affect the plant aboveground biomass after heterotroph removal. The presence of *B. striata* also increased the plant belowground biomass by 170.9% and 174.2%, respectively, under control and fungicide treatments (Table 2).

### 2.3. Functional Traits of Plant Leaves and Roots Respond to N Addition and Heterotroph Removal

**Figure 3:**
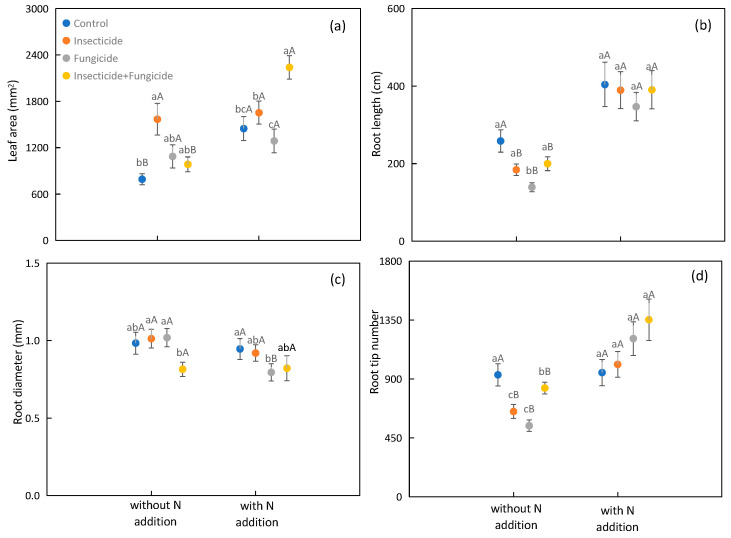
Difference in plant functional traits of plant leaf area (**a**), root length (**b**), root diameter (**c**), and root tip number (**d**) among heterotroph removal with or without N addition. Significant differences between systems without or with nitrogen addition were indicated in capital letters, and significant differences between heterotroph removals were indicated in lowercase letters. Each circle represents the average plant functional traits of all species compositions. Blue: control; orange: insecticide; gray: fungicide; yellow: insecticide and fungicide.

N addition increased the plant leaf area, root length, and root tip number by 49.5%, 95.9%, and 53.0% on average, respectively (Figure 3a,b,d). N addition increased the leaf area under control and all removal treatments (Figure 3a); N addition also increased the root length and root tip number in the insecticide, fungicide, and all removal treatment groups (Figure 3b,d); N addition reduced the root diameter under fungicide treatment (Figure 3c).

In systems without N addition, the insecticide treatment increased the leaf area by 98.1% relative to the control (Figure 3a). Fungicide treatment decreased the root length by 46.2% relative to the control (Figure 3b). Insecticide treatment increased the root diameter by 19.5%, and fungicide treatment increased the root diameter by 20.0% relative to the all removal treatment (Figure 3c). The insecticide, fungicide, and all removal treatments decreased the root tip number by 30.0%, 41.7%, and 10.9% relative to the control, respectively (Figure 3d). In systems with N addition, the all removal treatment increased the leaf area by 54.7%, and fungicide treatment decreased the root diameter by 16.0% relative to the control (Figure 3a,c). There were no significant differences found in the plant root length and tip number among heterotroph removal treatments (Figure 3b,d).

### 2.4. The Relationship between Plant Species Diversity and Functional Traits of Plant Leaves and Roots

Species richness significantly affected the plant leaf area and root tip number but did not affect the root length and diameter (Table S1). The plant leaf area decreased when the species richness increased to two and three, while the root tip number increased when species richness increased to three. Plant species compositions also significantly affected the plant leaf area, root length, root diameter, and root tip number (Table S1).

**Figure 4:**
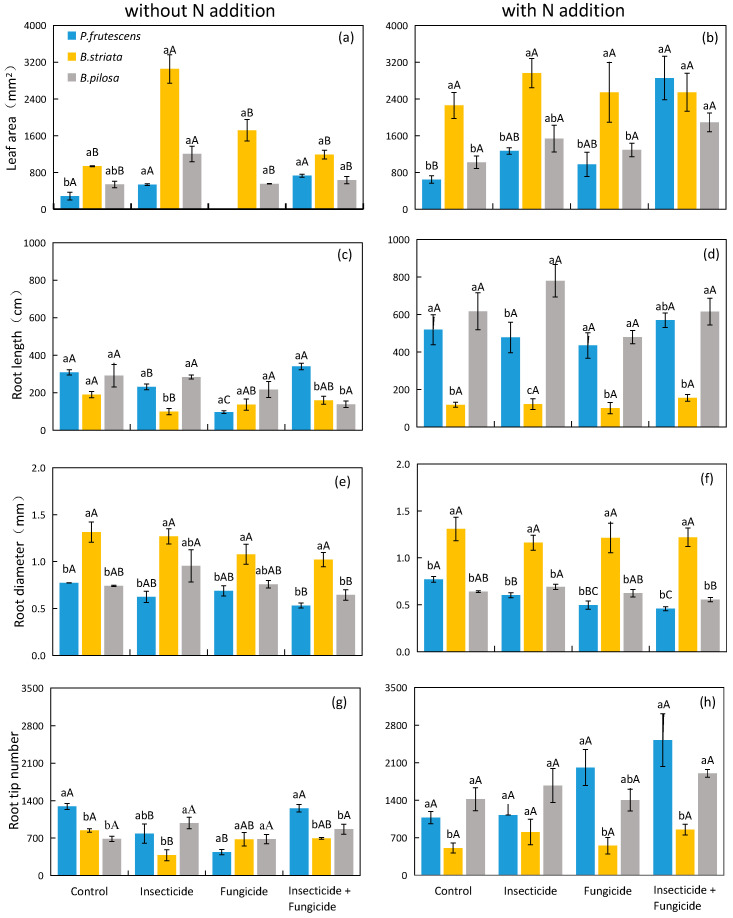
Difference in monoculture plant leaf area (**a**), root length (**c**), root diameter (**e**), root tip number (**g**) in systems without N addition and leaf area (**b**), root length (**d**), root diameter (**f**), root tip number (**h**) in systems with N addition among heterotroph exclusion with or without N addition. Significant differences between heterotroph removals were indicated in capital letters, and significant differences between plant species monocultures were indicated in lowercase letters. Blue bars: *P. frutescens* monoculture; yellow bars: *B. striata* monoculture; gray bars: *B. pilosa* monoculture.

In systems without N addition, the leaf area of the *B. striata* monoculture was significantly higher than that of the *P. frutescens* monoculture under control treatment groups (Figure 4a). However, the root diameter of the *B. striata* monoculture was significantly higher than that of the *P. frutescens* monoculture under each heterotroph removal treatment (Figure 4e), and the plant leaf area and root diameter were improved when *B. striata* was present (Table 3). The responses of plant root length and root tip number to the species identity were various under different heterotroph removal treatments. The presence of *B. striata* reduced the root tip number and root length of plants under control and insecticide treatments (Table 3), but the plant species identity did not affect root length under all heterotroph removal treatments.

In systems with N addition, the root length of the *B. striata* monoculture was significantly lower than that of the *B. pilosa* monoculture (Figure 4d), and the presence of *B. striata* reduced the root length and root tip number under each heterotroph removal treatment. The presence of *B. pilosa* increased the plant root length under each heterotroph removal treatment (Table 4). The response of plant root length to the species identity remained unchanged after heterotroph removal treatment. The root diameter of the *B. striata* monoculture was significantly higher than that of the *P. frutescens* monoculture and *B. pilosa* monoculture (Figure 4f), and the presence of *B. striata* increased the root diameter under each heterotroph removal treatment (Table 4).

## 3. Discussion

### 3.1. The Effect of Heterotroph Removal on Plant Biomass

**Figure 5:**
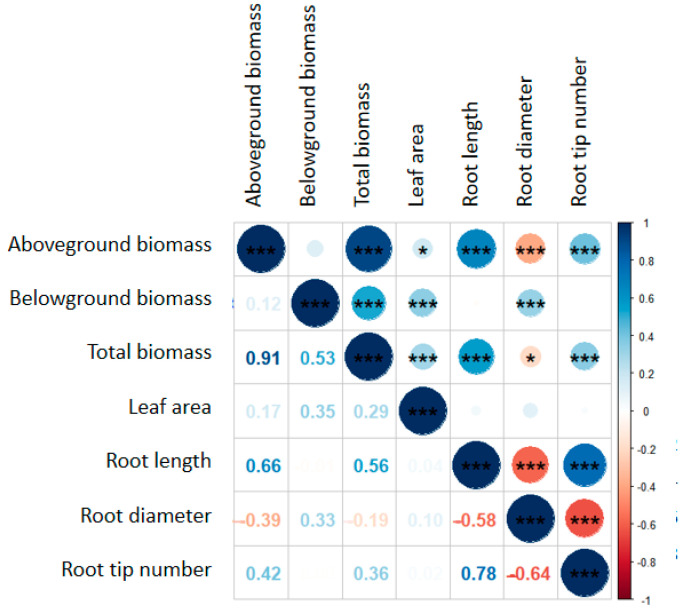
Correlation analysis between various parameters. * represents a significant relationship (*p* < 0.05), *** represents a significant relationship (*p* < 0.001).

Previous work found that heterotroph removal can increase plant biomass, and the effects of different heterotroph removals on plant biomass were different, with the highest increase in insecticide treatment groups [8,12,35]. In systems without N addition, the insecticide treatment increased the plant aboveground biomass by 99.0% and total biomass by 54.0%, relative to the control (Figure 1a). Meanwhile, the insecticide treatment increased the leaf area by 98.1% relative to the control (Figure 3a). According to the plant survival strategy, the plant biomass increased with the increase in plant leaf area [36]. We also found a positive correlation between the plant total biomass and leaf area (Figure 5). Insecticide treatment may increase plant biomass by increasing the plant leaf area. Another reason may be that arthropods have a negative impact on biomass [11]; thereby, insecticide treatment accumulates the biomass removed by herbivorous insects. Unlike previous research results, the plant biomass usually increases after fungicide treatment [11]; the plant aboveground and total biomasses did not increase, and even the plant belowground biomass decreased after fungicide treatment in this study (Figure 1b). Fungicide could remove some pathogens. However, some symbiotic bacteria in the soil that are beneficial for plant growth were affected by the fungicide treatment [9]. In addition, the plant root length and root tip number for fungicide treatment groups were lower than those under the control treatment (Figure 3b,d). These results indicated that heterotroph removal may influence the plant biomass by affecting the plant functional traits.

In systems with N addition, heterotroph removal did not affect the plant aboveground and total biomasses (Figure 1a,c). This result differs from those in systems without N addition (Figure 1a,c). N addition altered the effect of heterotroph removal on plant biomass. The possible reason may be that N addition provided sufficient environmental resources, increased plant N absorption, promoted photosynthesis, and increased plant biomass production [37], thereby reducing the effect of heterotroph removal on the plant biomass. The root system is the organ in which plants absorb nutrients from the soil, and roots can affect the plant biomass by affecting the soil nutrient turnover, nutrient utilization efficiency, and mycorrhizal infection. Heterotroph removal may affect the ecosystem function through plant functional traits [29,38,39]. In this study, there were no significant differences in the plant root length and diameter among heterotroph removal treatments in systems with N addition (Figure 3b,c). In addition, N addition may reduce the soil microbial community [40,41], further weakening the influence of heterotroph removal on plant biomass. These results indicated that the effect of heterotroph removal on plant biomass depends on the habitat N availability.

### 3.2. The Effect of Heterotroph Removal on Plant Species Richness–Biomass Relationship

Most research showed that plant productivity increased with the increase in plant species richness, and heterotroph removal altered the plant species diversity effect [4,8,12,42]. In the grassland system, removing the insecticide and fungicide treatments promoted the effect of plant species diversity on productivity [8]. However, in forest systems, a fungicide treatment eliminated the positive relationship between tree species richness and productivity [12]. In this study, the plant biomass did not respond to species richness under each heterotroph removal treatment in systems without N addition (Figure S1a,c,e). The reason may be that only three lower levels of richness were set in this experiment (1, 2, and 3), while most experiments were set to high levels of richness [9,14,43].

In systems with N addition, the impact of the plant species richness on plant aboveground and total biomasses was positive under all removal treatments (Figure S1b,f). High plant species richness improved the plant biomass through enhancing nutrient utilization [44], and N addition may promote this effect. Meanwhile, insecticide and fungicide treatments removed arthropods, leaf fungi, and soil fungi that could reduce plant biomass, causing a significant increase in the plant aboveground and total biomasses when species richness was three (Figure S1b,f). However, plant species richness had a negative impact on the plant belowground biomass under an insecticide treatment (Figure S3d). The reason may be that the abundance of arthropods increased with an increasing plant species richness [45], which may consume more plant leaf area. There was a positive correlation between the plant leaf area and belowground biomass (Figure 5), ultimately leading to a decrease in the plant belowground biomass with an increasing species richness under an insecticide treatment.

### 3.3. The Effect of Heterotroph Removal on the Effect of Plant Species Identity on Plant Biomass

Plant species identity is an important part of plant species diversity [1], and many studies have shown that the plant species identity affects plant biomass [42,46]. In systems without N addition, the presence of *B. striata* increased the plant belowground biomass under each heterotroph removal treatment (Table 1); the presence of *B. striata* also increased the plant leaf area and root diameter (Table 3). There was a significant positive correlation between the leaf area, root diameter, and plant belowground biomass (Figure 5). These results suggested that plant species identity affected the plant biomass by influencing the plant functional traits.

In systems with N addition, the presence of *B. striata* increased the plant belowground biomass in the control group, while this effect was dismissed after insecticide and fungicide treatments (Table 2). Meanwhile, the change pattern in the plant leaf area of *B. striata* was consistent with that of the plant belowground biomass (Table 4). Moreover, there was a significant positive correlation between the plant leaf area and plant belowground biomass (Figure 5), indicating that heterotroph removal altered the effect of species identity on the plant biomass by influencing the plant leaf area. In addition, N addition may change the interaction between plant species [26]. In this study, under insecticide or fungicide treatments, the relative yield of *B. striata* in systems with N addition was higher than that in systems without N addition (Figure S2). We also found that the selection effect became increasingly important in systems with N addition, resulting in an increase in the net biodiversity effect (Figure S3). Interestingly, the presence of *B. striata* decreased the plant aboveground biomass in the control group but did not affect the plant aboveground biomass after heterotroph removal treatment (Table 4). This study also found that the presence of *B. striata* increased the plant root diameter but decreased the plant root length and root tip number under each heterotroph removal treatment (Table 4). We observed a significant positive correlation between the plant root length, root tip number, and plant aboveground biomass, and a negative correlation between the root diameter and plant aboveground (Figure 5). These results also indicated that heterotroph removal changed the plant species identity’s effect on plant biomass by affecting the plant functional traits.

## 4. Materials and Methods

### 4.1. Experimental Design

**Figure 6:**
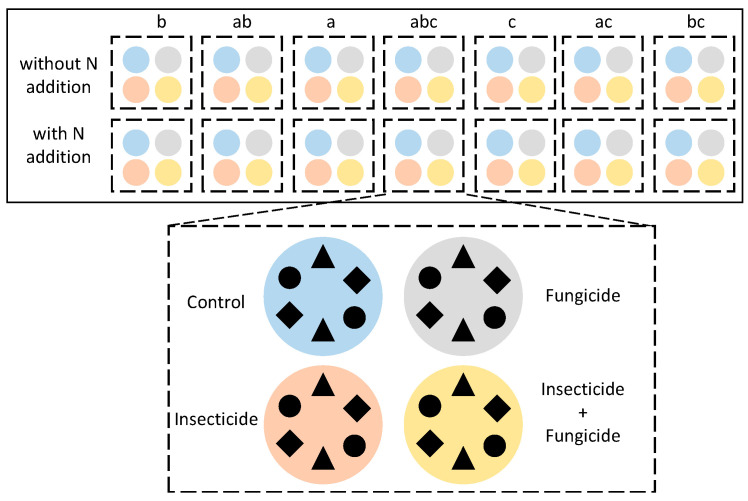
Experimental design (one block). The letters above the uppermost boxes represent plant species compositions, and species compositions within each group are randomly arranged. Colors represent heterotroph removal treatments: blue: control; orange: insecticide; gray: fungicide; yellow: insecticide and fungicide. The three-species treatment is depicted here, with different shapes representing different species. Six plants are planted in each experimental system uniformly.

The experiment was set up in a greenhouse at Wenzhou University in Wenzhou, Zhejiang Province, China (120°42′4″ E, 27°55′46″ N). The climate was a subtropical monsoon climate. The greenhouse has a transparent plastic roof, shielding the experiment from rainwater while maintaining temperature and humidity levels. A shading net was installed above the plastic roof to simulate the light environment under the forest. A three-factor control experiment was conducted (Figure 6): (1) species diversity: based on the functional trait, three local common understory herbaceous plants were chosen: *Perilla frutescens* (L.) *Britt*, *Bletilla striata*, and *Bidens pilosa* L. (Table S2) for plant species diversity configuration (all seven plant species compositions); (2) heterotroph removal treatments: control, insecticide, fungicide, and both insecticide and fungicide treatments; (3) N addition: N deposition was simulated by N addition, using without N addition as the control. There are four repetitions for each treatment. In total, 224 pots (30 cm diameter and 20 cm height) were constructed.

In April 2022, the seedlings of the plants were transplanted into pots, with six individuals planted in each pot. From the end of April to the beginning of September, heterotroph removal treatment and N addition treatment were conducted once a month. According to Seabloom et al. (2017), insects were removed by spraying an insecticide water emulsion (0.03% permethrin), fungi were removed by spraying fungicide (30% carbendazim), and the control group added an equal volume of water. Based on the environmental wet N deposition rate in Zhejiang Province (2.69 g N m^−2^ yr^−1^), we added ammonium nitrate (NH_4_NO_3_) solution every month to simulate high N conditions, with an average amount added each time (10 g N m^−2^ yr^−1^), and we added water as a control group.

Pesticides may impact plant growth even in the system without heterotrophs. Therefore, we designed a laboratory to test the impact of insecticides and fungicides on the plant biomass. The soil was homogenized and subjected to high-pressure steam treatment. Three plant species were treated with four heterotroph removal treatments, and each treatment had four replicates, totaling 48 pots with one individual in each pot. The heterotroph removal treatment was applied once a month, and the application amount was the same as the field experiment. Plants were allowed to grow for a total of ten weeks. After ten weeks, harvest each plant and divide it into aboveground and belowground biomasses. Results showed that heterotroph removal treatments do not affect plant biomass in the indoor experiment without consumers (Figure S4).

### 4.2. Sample Collection and Calculation

Plants were harvested at the end of the plant growth period. After washing harvested plants, three complete leaves and three roots were taken from each plant in each pot. After scanning with a scanner (EPSON GT-X980, Hangzhou, China), the images were analyzed and processed using the Wanshen leaf processing system (version 2018; www.Wseen.com) to obtain the leaf area, root length, root diameter, and number of root tips. Divide the plants into aboveground and belowground parts, dry at 105 °C for 20 min, then dry at 65 °C for 48 h to obtain each species’ aboveground and belowground biomass for each pot. The net effects, complementary effects, and selection effects were calculated according to Loreau and Hector’s calculation method [47]. The net effect refers to the difference between the observed yield (actual yield) and the expected yield (weighted average of individual yield corresponding to species in the mixture based on planting proportion) of the mixture. Complementary effects are measured from changes in the relative yield of species. The selection effect is measured by subtracting the complementary effect from the net effect.

1. Plant functional traits of leaves and roots:

To evaluate the response of plant functional traits to N addition and heterotroph removal treatments at the community level, the functional traits’ community weighted mean (CWM) was calculated:

(1) $$CWM=\sum_{i=1}^{s}E_{ic}\times B_{ic}$$

where E_ic_ represents species i’s plant functional traits in composition c, B_ic_ represents species i’s biomass in composition c (when calculating plant leaf area’s CWM, the B_ic_ referred to the proportion of species i’s aboveground biomass in composition c’s aboveground biomass; when calculating plant root traits’ CWM, the B_ic_ referred to the proportion of plant species i’s belowground biomass in composition c’s belowground biomass of composition c), and s referred to species’ amount in composition c.

2. Relative yield:

To evaluate whether N addition alter the competitiveness of specific species in the mixture, relative yield (RY) of the plant was calculated:

(2) $$RY_{i}=\frac{O_{i}}{E_{i}}$$

where *O*_*i*_ represents the aboveground biomass of species i per plant in the mixture, while *E*_*i*_ represents the aboveground biomass of species i per plant in the monoculture. If *RY*_*i*_ > 1, species i is the dominant species in the mixture.

### 4.3. Statistical Analysis

The influence of species diversity (species compositions and species richness), N addition, and heterotroph removal treatment on the plant biomass (aboveground, belowground, and total) and functional traits of plant leaves and roots was determined using a three-way ANOVA. The effects of plant species richness on plant biomass under each heterotroph removal treatment were tested using linear regression analysis. The difference in the above parameters between systems with and without N addition under the same heterotroph removal treatment was tested using an independent sample *t*-test. The effect of heterotroph removal treatment or plant species compositions on the above parameters under the same N habitat level was determined using a one-way ANOVA. If there were significant differences, the Tukey method was conducted. The effect of the plant species identity (the presence of certain species) on each parameter was determined using an independent sample *t*-test. The difference between the zero and net effects, complementary effects, and selection effects was examined using a single sample *t*-test. The correlations of various parameters were verified using Pearson’s correlation analysis. Before analysis, the data were ln-transformed to satisfy the equality of variance (Levene’s test) and assumptions of normality (Kolmogorov–Smirnov test). If the data after conversion still did not fulfill the assumptions, a nonparametric Kruskal–Wallis test was employed. All statistical analyses were conducted using the R4.1.1 program. All data were delivered as the mean ± standard error, and the statistical significance level was set as α = 0.05.

## 5. Conclusions

This study investigated the relationship between the plant species diversity and biomass response to heterotroph removal in systems with and without N addition. Our research findings indicate that heterotroph removal affected plant biomass by influencing the plant leaf area in both systems with or without N addition, and altered the effect of the plant species richness–plant biomass relationship by influencing the plant leaf area in systems with N addition but not in systems without N addition. Heterotroph removal also affected the effect of species identity on the plant biomass by influencing the plant functional traits in both systems with or without N addition. Therefore, it is recommended that in the background of global N deposition, the impact of other trophic level organisms on ecosystem functioning cannot be ignored when analyzing the species diversity–ecosystem functions relationship. In terms of ecosystem management, biodiversity at different trophic levels should be protected. In the future, more high plant species diversity experiments with long-term research are needed to determine the impact of heterotrophs on biodiversity–ecosystem function relationships in high-N habitats.

## Figures and Tables

**Table 1:** Plant biomass (aboveground, belowground, and total) responses to species identity without N addition; *p* values are displayed in bold font when *p* < 0.05.

Source of Variation	Aboveground Biomass		Belowground Biomass		Total Biomass	
	*p* Value	Change	*p* Value	Change	*p* Value	Change
**Control**						
*P. frutescens*	0.223	ns	0.135	ns	0.061	ns
*B. striata*	0.357	ns	**<0.001**	↑ 381.67%	0.058	ns
*B. pilosa*	**<0.001**	↑ 343.78%	0137	ns	0.818	ns
**Insecticide**						
*P. frutescens*	0.121	ns	0.109	ns	**0.003**	↓ 49.72%
*B. striata*	0.942	ns	**<0.001**	↑ 356.69%	0.071	ns
*B. pilosa*	**<0.001**	↑ 300.77%	0.216	ns	0.189	ns
**Fungicide**						
*P. frutescens*	0.554	ns	0.152	ns	0.126	ns
*B. striata*	0.102	ns	**<0.001**	↑ 252.87%	0.645	ns
*B. pilosa*	**<0.001**	↑ 472.00%	1.000	ns	**0.001**	↑ 110.19%
**Insecticide + Fungicide**						
*P. frutescens*	0.567	ns	0.056	ns	0.144	ns
*B. striata*	0.397	ns	**0.003**	↑ 138.16%	0.765	ns
*B. pilosa*	**<0.001**	↑ 506.20%	0.635	ns	**<0.001**	↑ 155.78%

Notes: Values and arrows in ‘Change’ column show significant increase (↑) or decrease (↓) of the variables with the presence of a certain species compared to its absence, and ‘ns’ means no significant change.

**Table 2:** Plant biomass (aboveground, belowground, and total) responses to species identity with N addition; *p* values are displayed in bold font when *p* < 0.05.

Source of Variation	Aboveground Biomass		Belowground Biomass		Total Biomass	
	*p* Value	Change	*p* Value	Change	*p* Value	Change
**Control**						
*P. frutescens*	0.235	ns	0.487	ns	0.095	ns
*B. striata*	**0.016**	↓ 58.09%	**<0.001**	↑ 170.89%	0.357	ns
*B. pilosa*	**<0.001**	↑ 735.36%	**0.040**	↓ 46.65%	**0.002**	↑ 122.13%
**Insecticide**						
*P. frutescens*	0.247	ns	**0.002**	↓ 75.01%	**0.009**	↓ 52.30%
*B. striata*	0.111	ns	0.178	ns	0.114	ns
*B. pilosa*	**<0.001**	↑ 425.05%	0.516	ns	**<0.001**	↑ 220.75%
**Fungicide**						
*P. frutescens*	0.316	ns	0.134	ns	0.069	ns
*B. striata*	0.464	ns	**0.018**	↑ 174.19%	0.555	ns
*B. pilosa*	**<0.001**	↑ 270.44%	0.237	ns	**0.003**	↑ 85.24%
**Insecticide + Fungicide**						
*P. frutescens*	0.432	ns	**0.014**	↓ 60.88%	0.980	ns
*B. striata*	0.077	ns	0.236	ns	0.148	ns
*B. pilosa*	**<0.001**	↑ 355.43%	0.347	ns	**<0.001**	↑ 211.94%

Notes: Values and arrows in ‘Change’ column show significant increase (↑) or decrease (↓) of the variables with the presence of a certain species compared to its absence, and ‘ns’ means no significant change.

**Table 3:** Functional traits of plant leaves and roots respond to species identity without N addition; *p* values are displayed in bold font when *p* < 0.05.

Source of Variation	Leaf Area		Root Length		Root Diameter		Root Tip Number	
	*p* Value	Change	*p* Value	Change	*p* Value	Change	*p* Value	Change
**Control**								
*P. frutescens*	0.136	ns	0.063	ns	0.208	ns	**0.007**	↑ 62.01%
*B. striata*	**<0.001**	↑ 95.19%	**0.002**	↓ 52.62%	**0.012**	↑ 51.69%	**0.016**	↓ 37.89%
*B. pilosa*	0.133	ns	0.341	ns	**0.037**	↓ 25.67%	0.138	ns
**Insecticide**								
*P. frutescens*	0.123	ns	0.311	ns	**0.035**	↓ 21.59%	0.480	ns
*B. striata*	**0.006**	↑ 96.78%	**<0.001**	↓ 40.80%	**<0.001**	↑ 53.61%	**0.010**	↓ 34.62%
*B. pilosa*	**0.004**	↓ 57.27%	0.235	ns	0.494	ns	0.589	ns
**Fungicide**								
*P. frutescens*	0.177	ns	0.180	ns	0.255	ns	**0.032**	↓ 25.80%
*B. striata*	**0.002**	↑ 129.53%	0.413	ns	**<0.001**	↑ 59.98%	0.432	ns
*B. pilosa*	0.368	ns	**0.036**	↑ 41.28%	0.619	ns	0.378	ns
**Insecticide + Fungicide**								
*P. frutescens*	0.744	ns	0.119	ns	0.268	ns	0.052	ns
*B. striata*	**0.002**	↑ 81.35%	0.135	ns	**<0.001**	↑ 51.78%	**0.039**	↓ 21.53%
*B. pilosa*	0.275	ns	0.876	ns	0.109	ns	0.445	ns

Notes: Values and arrows in ‘Change’ column show significant increase (↑) or decrease (↓) of the variables with the presence of a certain species compared to its absence, and ‘ns’ means no significant change.

**Table 4:** Functional traits of plant leaves and roots respond to species identity with N addition; *p* values are displayed in bold font when *p* < 0.05.

Source of Variation	Leaf Area		Root Length		Root Diameter		Root Tip Number	
	*p* Value	Change	*p* Value	Change	*p* Value	Change	*p* Value	Change
**Control**								
*P. frutescens*	0.111	ns	0.542	ns	0.693	ns	0.913	ns
*B. striata*	**0.025**	↑ 55.64%	**0.007**	↓ 53.30%	**<0.001**	↑ 73.57%	**<0.001**	↓ 48.59%
*B. pilosa*	0.762	ns	**<0.001**	↑ 225.29%	**<0.001**	↓ 36.05%	**0.001**	↑ 90.01%
**Insecticide**								
*P. frutescens*	**0.023**	↓ 34.55%	0.865	ns	0.077	ns	0.678	ns
*B. striata*	0.090	ns	**0.008**	↓ 64.61%	**<0.001**	↑ 67.27%	**<0.001**	↓ 41.13%
*B. pilosa*	0.664	ns	**0.018**	↑ 79.98%	0.900	ns	0.667	ns
**Fungicide**								
*P. frutescens*	0.180	ns	0.824	ns	0.406	ns	0.826	ns
*B. striata*	0.832	ns	**0.014**	↓ 36.42%	**<0.001**	↑ 62.31%	**0.048**	↓ 34.31%
*B. pilosa*	0.781	ns	**0.003**	↑ 94.93%	**0.046**	↓ 27.22%	0.239	ns
**Insecticide + Fungicide**								
*P. frutescens*	0.273	ns	0.432	ns	0.252	ns	0.622	ns
*B. striata*	0.810	ns	**<0.001**	↓ 64.46%	**<0.001**	↑ 90.17%	**<0.001**	↓ 57.71%
*B. pilosa*	**0.026**	↓ 26.22%	**0.015**	↑ 76.82%	0.164	ns	0.180	ns

Notes: Values and arrows in ‘Change’ column show significant increase (↑) or decrease (↓) of the variables with the presence of a certain species compared to its absence, and ‘ns’ means no significant change.

## Data Availability

Data are available on request from the authors. The data are not publicly available due to [Some data are acquired from other collaborators].

## Supplementary Materials

The following supporting information can be downloaded at: https://www.mdpi.com/article/10.3390/plants13020258/s1, Table S1: Three-way ANOVA Table (a) Effects of nitrogen addition, species compositions, heterotrophs removal treatment, and (b) Effects of nitrogen addition, species richness, and heterotrophs removal treatment on above-, below-ground, total biomass and functional traits of plant leaves and roots; Table S2: The plant functional traits of *Perilla frutescens*, *Bletilla striata* and *Bidens pilosa*; Figure S1. Linear regression of species richness on plant aboveground biomass (a), belowground biomass (c), and total biomass (e) in systems without N addition and plant aboveground biomass (b), belowground biomass (d), and total biomass (f) in systems with N addition under different heterotrophs removal treatment. Figure S2. The relative yield of *B. pilosa* under different heterotroph treatments and N availability. Significant differences between heterotroph removal were indicated in capital letters, and significant differences between N availability were indicated in lowercase letters. Blue circle: without N addition; yellow, with N addition. Figure S3. Net effect of aboveground biomass (a), belowground biomass (b), and total biomass (c); complementary effect of aboveground biomass (d), belowground biomass (e), and total biomass (f); selection effect of aboveground biomass (g), belowground biomass (h), and total biomass (j) under different heterotroph removal treatments. * Represents a significant effect under this treatment. Blue bar: control; orange, insecticide; gray, fungicide; yellow, insecticide and fungicide. Figure S4. Aboveground (a), belowground (b), and total biomass (c) of plant monoculture under different heterotrophs treatments. Same lowercase letters indicate no difference between het-erotrophs removal. Blue bar: control; orange, insecticide; gray, fungicide; yellow, insecticide and fungicide.
