# Supplementary material for: Nitrogen Addition and Heterotroph Exclusion Affected Plant Species Diversity–Biomass Relationship by Affecting Plant Functional Traits
Source: Plants (Basel). 2024 Jan 16;13(2):258. doi: 10.3390/plants13020258 (PMC10818353; doi:10.3390/plants13020258)
Supplement: Supplementary file 1 [file plants-13-00258-s001.zip › plants-2737127-supplementary.pdf]

plants

MDPI

Table S1. Three-way ANOVA Table (a) Effects of nitrogen addition, species compositions, heterotrophs removal treatment, and (b) Effects of nitrogen addition, species richness, and heterotrophs removal treatment on above-, below-ground, total biomass and functional traits of plant leaves and roots.

Variables	Aboveground biomass	Belowground biomass	Total biomass	Leaf area	Root length	Root diameter	root tip number
（a）							
NA	**<0.001**	**<0.001**	**<0.001**	0.861	**<0.001**	**0.002**	**<0.001**
SC	**<0.001**	**<0.001**	**<0.001**	**<0.001**	**<0.001**	**<0.001**	**<0.001**
HR	0.750	**<0.001**	0.057	0.356	**0.013**	**0.002**	0.125
NA×SC	**<0.001**	**0.015**	**<0.001**	**<0.001**	**<0.001**	0.584	**<0.001**
NA×HR	0.539	0.591	0.483	0.952	0.389	0.847	**<0.001**
SC×HR	**0.010**	0.103	**0.028**	0.374	**0.030**	0.889	0.062
NA×SC×HR	**0.001**	0.485	**0.020**	0.424	**0.003**	0.602	**0.032**
（b）							
NA	**<0.001**	**<0.001**	**<0.001**	0.655	**<0.001**	**0.043**	**<0.001**
SR	**0.038**	0.177	0.386	**0.004**	0.264	0.147	**0.009**
HR	0.699	**<0.001**	0.177	0.643	0.196	**0.016**	0.125
NA×SR	0.057	**0.015**	0.783	0.249	0.753	0.470	0.266
NA×HR	0.689	0.527	0.525	0.649	0.524	0.935	**0.003**
SR×HR	0.102	0.366	0.069	0.275	0.769	0.328	0.358
NA×SR×HR	0.188	0.569	0.155	0.983	0.787	0.361	0367

[1] Notes: NA represents Nitrogen addition, SC represents Species composition, SR represents Species richness, HR represents Heterotropic removal treatment. Cells with significant impact are displayed in bold.

Table S2. The plant functional traits of *Perilla frutescens*, *Bletilla striata* and *Bidens pilosa*

Plants	Height (cm)	Basal diameter (mm)	Leaf area (mm^2)	Root length (cm)	Root diameter (mm)	root tip number
P. frutescens	18.1	2.7	285.5	308.7	0.773	1291
B. striata	30.1	1.3	936.7	190.1	1.314	843
B. pilosa	39.7	2.7	538.9	290.5	0.741	686

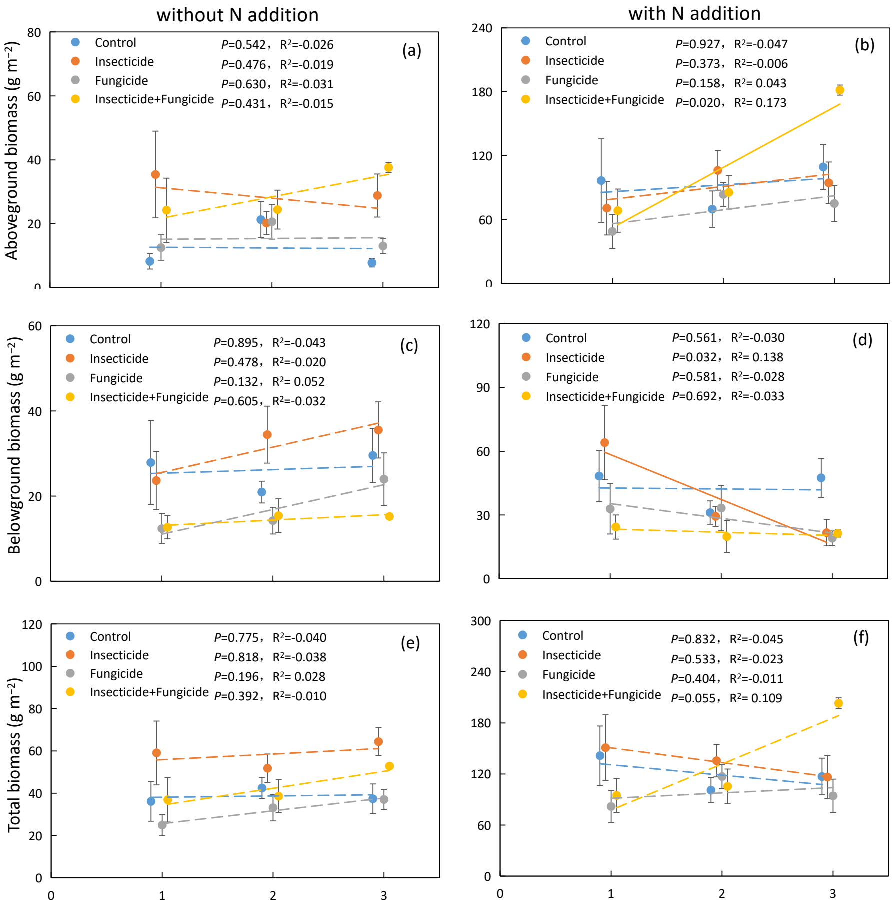

Figure S1. Linear regression of species richness on plant aboveground biomass (a), belowground biomass (c), and total biomass (e) in systems without N addition and plant aboveground biomass (b), belowground biomass (d), and total biomass (f) in systems with N addition under different heterotrophs removal treatment.

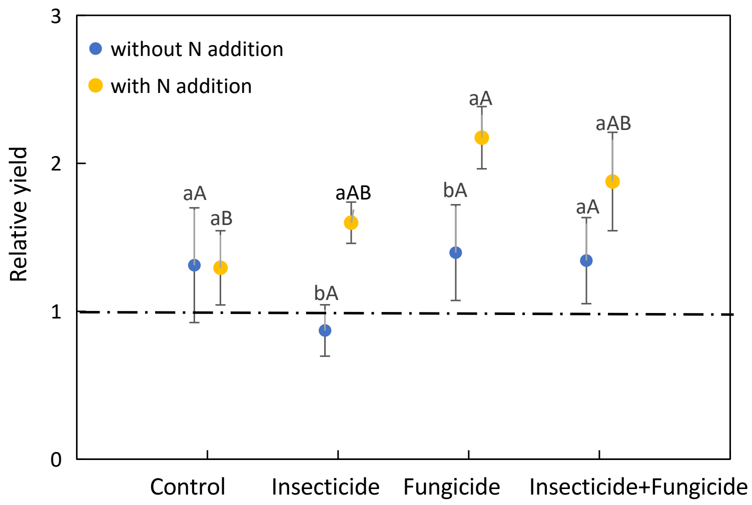

Figure S2. The relative yield of *B. pilosa* under different heterotroph treatments and N availability. Significant differences between heterotroph removal were indicated in capital letters, and significant differences between N availability were indicated in lowercase letters. Blue circle: without N addition; yellow, with N addition.

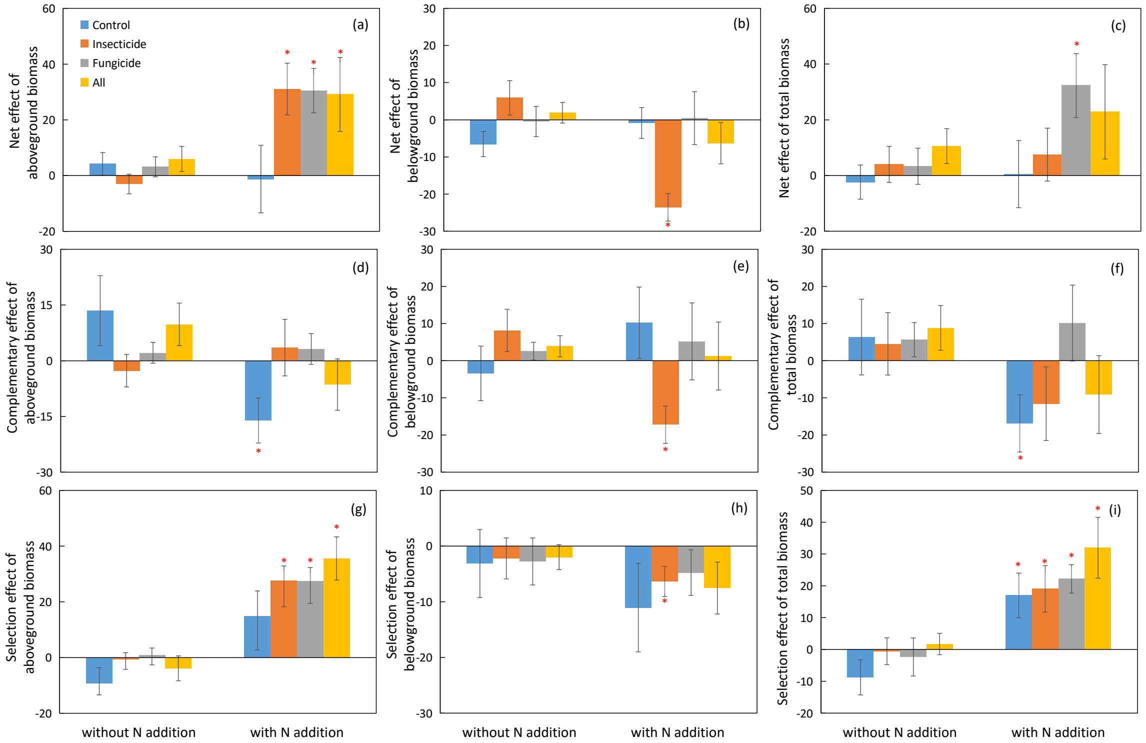

Figure S3. Net effect of aboveground biomass (a), belowground biomass (b), and total biomass (c);, complementary effect of aboveground biomass (d), belowground biomass (e), and total biomass (f); selection effect of aboveground biomass (g), belowground biomass (h), and total biomass (j) under different heterotroph removal treatments. *Represents a significant effect under this treatment. Blue bar: control; orange, insecticide; gray, fungicide; yellow, insecticide and fungicide.

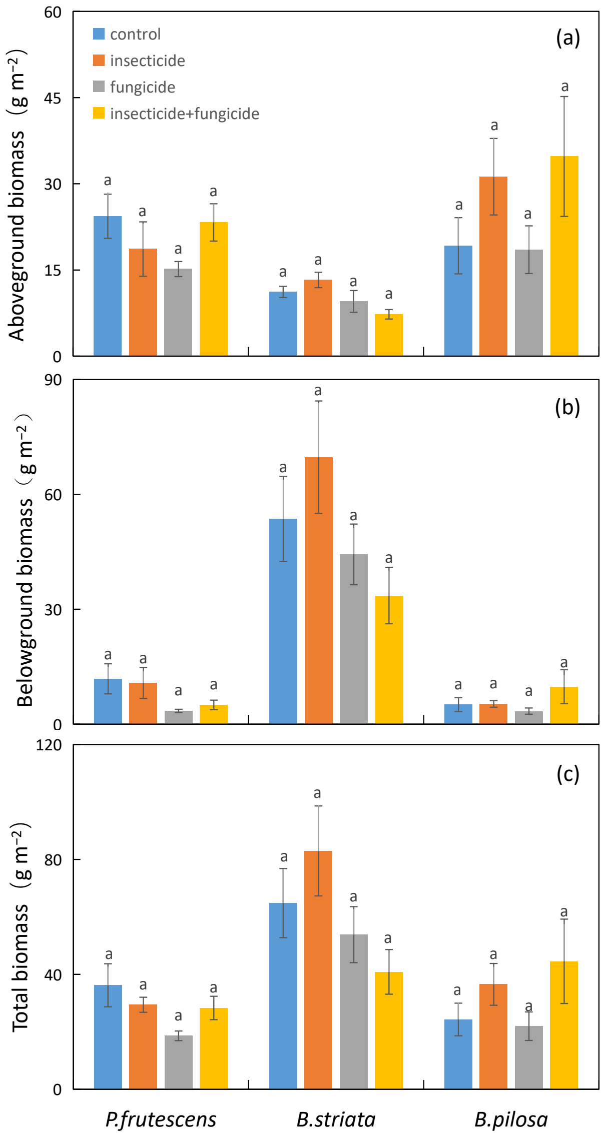

Figure S4. Aboveground (a), belowground(b), and total biomass (c) of plant monoculture under different heterotrophs treatments. Same lowercase letters indicate no difference between heterotrophs removal. Blue bar: control; orange, insecticide; gray, fungicide; yellow, insecticide and fungicide.
